# Tracheotomy Outcomes in 71 COVID-19 Patients: A Multi-Centric Study in Saudi Arabia

**DOI:** 10.3390/clinpract11040109

**Published:** 2021-12-15

**Authors:** Dakheelallah Almutairi, Raneem Alqahtani, Arwa Alghamdi, Dina Binammar, Suzan Alzaidi, Abdullah Ghafori, Hassan Alsharif

**Affiliations:** 1 Department of Otorhinolaryngology-Head and Neck Surgery, King Abdulaziz Medical City, Jeddah 21423, Saudi Arabia; Drmutairi1@gmail.com; 2 College of Medicine, King Saud Bin-Abdul-Aziz University for Health Sciences, Ministry of National Guard, Jeddah 14611, Saudi Arabia; Arwa.Alghamdi1996@gmail.com (A.A.); Dina.y.ammar@gmail.com (D.B.); 3 Department of Otorhinolaryngology-Head and Neck Surgery, King Fahad Armed Forces Hospital, Jeddah 23311, Saudi Arabia; alzaidisuzan@gmail.com (S.A.); dr_ghafouri2006@me.com (A.G.); 4 Intensive Care Department (ICD), King Fahad Armed Forces Hospital, Jeddah 23311, Saudi Arabia; alsharif_hassan@yahoo.ca

**Keywords:** COVID-19, tracheostomy, intubation, ICU, mechanical ventilation

## Abstract

Since its outbreak in late 2019, the COVID-19 pandemic has seen a sharp rise in the demand for oxygen and ventilation facilities due to the associated extensive damage that it causes to the lungs. This study is considered the first and largest study in Saudi Arabia to evaluate the outcomes of tracheostomy in intubated COVID-19 patients. This is a retrospective, observational cohort study that was conducted at King Abdulaziz Medical City (KAMC) in Jeddah, Western Region, Saudi Arabia and King Fahad Armed Forces Hospital, Jeddah, Saudi Arabia. The findings of the study revealed that seventy-one patients with COVID-19 underwent tracheotomy between 1 March 2020 and 31 October 2020. The average period between intubation and tracheostomy was 9.97 days. Hypertension, diabetes, lung disease and obesity (BMI > 30) were significant risk factors of mortality. The overall 30-day mortality rate was 38.4%.

## 1. Introduction

Since its identification in late 2019, COVID-19 disease, caused by SARS-CoV-2, has continued to spread across the world, causing a remarkable global healthcare burden. To date, reports from Saudi Arabia have revealed 548,000 confirmed cases and almost 8000 deaths, forfeiting the country’s advanced place in terms of effectively combating the disease [1].

Acute respiratory distress (ARDS) is the hallmark of COVID-19 disease, with a reported incidence rate of 17–19% in hospitalized COVID-19 patients, increasing the demand on mechanical ventilation and intensive care unit (ICU) beds and likely jeopardizing healthcare recourses. As per the previous reports, 2.3–15.2% of COVID-19 patients eventually require mechanical ventilation [2,3,4].

Tracheostomy should always be considered when a prolonged need for mechanical ventilation is anticipated. It comprises two techniques: open surgical tracheostomy and percutaneous tracheostomy; however, the literature reports no significant difference in the rate of major complications between the two in non-COVID-19 patients. When compared to endotracheal intubation, tracheostomy ensures a lower need for sedation, decreased work for breathing, better patient communication, improved weaning and, most importantly, a reduced risk of laryngeal and tracheal stenosis. However, the paucity of evidence of tracheostomy outcomes and its optimal timing in COVID-19 patients may limit its use [4,5,6]. As tracheostomy is considered an aerosol-generating procedure (AGP) that increases the risk of viral exposure and transmission, the international guidelines suggest delaying its use among intubated patients with COVID-19 [7]. The optimal timing is still a controversial issue. The American Academy of Otolaryngology-Head and Neck Surgery (AAO-HNS) recommends delaying the timing for at least 14 days to decrease the risk of transmission of the virus [4]. However, early tracheostomy may be urgently indicated in life-threatening situations in which a tracheostomy would significantly increase the survival rate, including patients with upper airway obstruction with the inability to intubate [7].

Several studies have investigated the early outcomes of tracheostomy in COVID-19 patients, most of which have shown high rates of successful decannulation and liberation from ventilation and low rates of mortality and major complications [4,8,9]. Reviewing the literature, tracheostomy outcomes in COVID-19 patients have never been reported in Saudi Arabia.

## 2. Materials and Methods

### 2.1. Study Setting and Participants

This is a retrospective, observational cohort study that was conducted at King Abdulaziz Medical City (KAMC) in Jeddah, Western Region, Saudi Arabia and King Fahad Armed Forces Hospital, Jeddah, Saudi Arabia. All COVID-19 intubated patients in intensive care units (ICU) aged above 18 years old who underwent either an open tracheostomy that was performed by an expert otolaryngologist or a percutaneous tracheostomy that was performed by an ICU consultant between 1 March 2020 and 31 October 2021 were included in our study. Nasal polymerase chain reaction (PCR) was used to confirm the diagnosis of COVID-19 in all patients. 

### 2.2. Data Collection

Using a self-administered data collection sheet, the medical records were reviewed for demographic information, medical comorbidities, length of hospital stay and related events, such as days from admission to tracheostomy, length of ICU stay, days from intubation to tracheostomy, days from tracheostomy to ICU and hospital discharge. The tracheostomy-related parameters, such as the type of tracheostomy, timing of tracheostomy, complications and mechanical ventilation history, were also abstracted.

### 2.3. Statistics and Data Analysis

The researchers entered the data into a workplace computer and analyzed it using SPSS (Statistical Package Social Sciences) version 26 (Armonk, NY, USA, 2019). All data files were given a serial number, omitting the names and personal details. The information and data were saved and stored digitally in a password-protected work-based computer, which was only accessed by the investigators. The Shapiro–Wilk test was used to assess the normality. Normally distributed data were presented as the mean and standard deviation (SD), and non-normally distributed data were presented as the median and interquartile range (IQR). Categorical variables were presented as the count and percentage. For categorical variables, the chi-square test was used, and the *t*-test was used for continuous variables. A *p*-value < 0.05 was considered significant. A multivariate logistic regression analysis was used to control the possible cofounder variables and to identify the most predictive factors associated with mortality in tracheostomized patients. The results were expressed as the odds ratio (OR) and 95% CIs.

### 2.4. Ethics

Approval from the Regional Research and Ethics Committee at King Abdullah International Medical Research Center Western Region (NRJ21J/142/05) and King Fahd Armed Forces Hospital-Jeddah Research and Ethics Committee (REC 443) was obtained. Ethical considerations were taken into account throughout all the research steps. Due to the retrospective nature of the investigation, informed consent was not required.

## 3. Results

### 3.1. Demographic Characteristics and Comorbidities of the Study Participants

In this study, 71 COVID-19 patients underwent a tracheostomy from 1 March 2020 to 31 January 2021. The target population was aged between 21 and 84 years, with the median age being 60 years (IQR = 15). Out of this sample, 47 (66.2%) were male, while 24 (33.8%) were female. Furthermore, 56 (78.9%) of the participants underwent percutaneous tracheotomy, and 15 (21.1%) underwent open tracheotomy. The most common coexisting condition was diabetes (53.5%, 38 out of 71), followed by hypertension (45.1%, 32 out of 71), obesity (36.6%, 26 out of 71), hyperlipidemia (35.2%, 25 out of 71), lung disease (31%, 22 out of 71) and heart disease (26.4%, 18 out of 71). The demographic characteristics of the study population are summarized in Table 1.

### 3.2. Timing of Tracheostomy Events

The average duration between hospital admission and tracheostomy was 13.32 ± 2.63 days. The mean period between intubation and tracheostomy was 9.97 ± 2.72 days. The average days of ICU stay before tracheostomy were 10.90 ± 2.4 days. The average days between tracheostomy and weaning from the ventilator were 6.39 ± 1.53 days, while patients took an average of 13.68 ± 2.91 days to be discharged from the ICU after a tracheostomy. The timing of the tracheostomy events is summarized in Table 2.

### 3.3. Prognostic Risk Factors and Mortality after Tracheostomy

In our study, hypertension, diabetes, lung disease and obesity (BMI > 30) were significant risk factors of mortality (*p*-values of 0.033 (OR = 0.34), 0.015 (OR = 0.28), 0.023 (OR = 0.30) and 0.17 (OR = 0.29), respectively). No significant differences were observed among survivors and non-survivors in terms of age, gender, hyperlipidemia and heart disease (*p*-values of 0.536 (OR = 0.58), 0.075 (OR = 2.64), 0.276 (OR = 0.72) and 0.105 (OR = 0.41), respectively). The association between various prognostic factors and mortality after tracheostomy is summarized Table 3. 

### 3.4. Tracheotomy Complications and Outcomes

Over the duration of the study, the 30-day mortality rate was 38.4% (28 out of 71). In terms of COVID-19 tracheostomy complications, the most common complication was oozing from the tracheostomy site (15.5%, 11 out of 71), followed by tracheostomy tube obstruction by a mucous plug (7%, 5 out of 71), a positional cuff leak (4.2%, 3 out of 71), tracheal infection (4.2%, 3 out of 71) and pneumomediastinum (2.8%, 2 out of 71). The statistics on the COVID-19 tracheostomy complications and outcomes are presented in Table 4.

### 3.5. Tracheostomy-Related Events between Survivors and Non-Survivors

The average number of days from tracheostomy to ICU discharge among the survivors was significantly higher (14.56 ± 3.69) than the mean number of days from tracheostomy to death among non-survivors (12.32 ± 2.35), with a *p*-value of 0.044. On the contrary, the number of days from hospital admission to tracheostomy, intubation to tracheostomy, ICU admission to tracheostomy and tracheostomy to weaning from the ventilator carried no significance between survivors and non-survivors, as indicated in Table 5.

## 4. Discussion

Since its outbreak in late 2019, the COVID-19 pandemic has seen a sharp rise in the demand for oxygen and ventilation facilities due to the associated extensive damage that it causes to the lungs. Considering the heavy burden of the pandemic on the global healthcare sector, tracheostomy is one of the most effective interventions that can be used to bridge the ventilation needs of COVID-19 patients. Other advantages of this procedure include facilitating the early ventilator weaning of COVID-19 patients, decreasing the duration of ICU stay and freeing up ICU resources for patients who may be recovering from conditions other than COVID-19.

Despite the outlined advantages, there are challenges, such as the severity of the COVID-19 infection and technical difficulties in performing the procedure, both of which are stumbling blocks to realizing the full benefits of tracheostomy among COVID-19 patients. In this paper, we aimed to broaden our knowledge and to bridge the gap in the recent literature on optimal timing and outcomes in COVID-19 patients after undergoing tracheostomy. To date, our study represents the largest series of tracheostomies performed among COVID-19 patients in Saudi Arabia, and it covers our experience performing tracheostomies on 71 patients during the peak of the pandemic in our country. According to our findings, patients who underwent tracheostomy were predominantly males (66.2%) as compared to females (33.8%). This is in accordance with previous studies that have reported a higher disease severity and poor prognosis in males [9,10].

The timing of tracheostomy among COVID-19 patients is still controversial, and several guidelines on this topic have not yet reached a consensus. Earlier recommendations encouraged postponing tracheostomy beyond 14 days, when the danger of viral transmission is minimal and the prognosis of the critically ill patient becomes clearer [9,11,12,13]. Moreover, early tracheostomy makes prone invasive ventilation difficult in the early course of the disease, and patients with severe hypoxemia will not tolerate the loss of positive airway pressure during the procedure [14,15]. Additionally, a Chinese multi-center study found that tracheostomies performed within 14 days of intubation were linked to a higher death risk than those performed after 14 days [16]. On the contrary, a prospective cohort analysis study of 50 patients showed that early tracheostomy (<10 days) is not associated with death [17]. Moreover, a recent systematic review and meta-analysis of non-COVID 19 patients found that tracheostomy performed early (within 7 days) was found to have a lower incidence of mortality and ventilator-associated pneumonia, as well as a shorter period of mechanical ventilation and ICU stay [18,19]. In our study, the mean period of intubation before a tracheostomy was 9.97 ± 2.72 days. A recent study by Chandran A et al. reported a mean of 10.27 days from intubation to tracheostomy [20]. Another study by Chao TN et al. reported an average time of intubation before a tracheostomy of 19.7 ± 6.9 days, with a range of 8–42 days [9].

The mortality among COVID-19 patients who underwent tracheostomy in previous published studies ranged from 7% to 41% [9,21]. Moreover, a study by Chandran A et al. had an overall 30-day mortality of 66.66% [20]. In this study, the overall 30-day mortality rate was 38.4%, which is an acceptable rate in comparison to the previously reported cases.

Multiple medical comorbidities, such as hypertension, diabetes, lung disease and obesity (BMI > 30), were associated with poor outcomes. Previous studies found that the presence of sepsis, older age and coexisting neurological diseases were associated with poor outcomes [20,22]. Moreover, a study by Chandran A et al. found an overall 30-day mortality rate of 66.66% [20]. In this study, the overall 30-day mortality rate was 38.4%, which is acceptable in comparison to previously reported cases.

In our series, the most common complication was oozing from the tracheostomy site (15.5%, 11 out of 71), followed by the mucous plug (7%, 5 out of 71), positional cuff leak (4.2%, 3 out of 71), tracheal infection (4.2%, 3 out of 71) and pneumomediastinum (2.8%, 2 out of 71). Moreover, a study by Ahmed Y et al. reported that the incidence of tracheostomy-related complications was 19% [4].

## 5. Conclusions

This study was one of the first in Saudi Arabia to describe the outcomes of tracheostomy in COVID-19 patients. In accordance with the literature, our data showed that tracheostomy is an effective weaning tool with acceptable complication and mortality rates.

## 6. Limitations

This study identified tracheostomy in COVID-19 patients as an effective and safe weaning tool; however, there were some major limitations that could be addressed in future research: (1) the small sample size, (2) the study design and (3) the inconsistent reporting of data in the medical charts, which might be explained by the pandemic burden and the limited personnel and resources.

## Figures and Tables

**Table 1:** Patient demographics, comorbidities and tracheotomy summary data.

Patient Number (*n* = 71)	
Age (median (IQR); range)	60 (15); 21–84
Male sex, No. (%)	47 (66.2)
**Coexisting Conditions**	**No. (%)**
Hypertension	32 (45.1)
Hyperlipidemia	25 (35.2)
Diabetes	38 (53.5)
Lung Disease	22 (31)
Heart disease	18 (25.4)
Obesity (BMI ≥ 30 kgm^−2^)	26 (36.6)
**Tracheostomy Technique**	**No. (%)**
Percutaneous	56 (78.9)
Open	15 (21.1)

**Table 2:** The timing of the tracheostomy events.

Tracheostomy Events (days)	Mean ± SD
Hospital admission to tracheostomy	13.32 ± 2.63
Intubation to tracheostomy	9.97 ± 2.72
ICU admission to tracheostomy	10.90 ± 2.4
Tracheostomy to weaning from the ventilator	6.39 ± 1.53
Tracheostomy to ICU discharge	13.68 ± 2.91

**Table 3:** The association between the prognostic risk factors and mortality after tracheostomy.

Prognostic Variable	Category	Number of Patients		*p*	OR *	95% CI **
		Non-Survivors Survivors				
Age	More than 40 years	2	5	0.536	0.58	0.10–3.24
	More than 40 years	26	38			
Gender	Male	2	25	0.075	2.64	0.89–7.83
	Female	6	18			
Hypertension	Yes	17	15	0.033	0.34	0.13–0.92
	NO	11	28			
Hyperlipidemia	Yes	12	13	0.276	0.72	0.21–1.55
	NO	16	30			
Diabetes	Yes	20	18	0.015	0.28	0.10–0.79
	NO	8	25			
Lung Disease	Yes	13	9	0.023	0.30	0.10–0.86
	NO	15	34			
Heart Disease	Yes	10	8	0.105	0.41	0.13–1.22
	NO	18	35			
BMI, subgroups	<30	13	32	0.017	0.29	0.10–2.73
	>30	15	11			

* Odds ratio (OR) and ** confidence interval (CI).

**Table 4:** COVID-19 tracheostomy complications and outcomes.

Complications	Number (%)
Oozing from Tracheostomy site	11 (15.5)
Mucous plug	5 (7)
Positional cuff leak	3 (4.2)
Pneumomediastinum	2 (2.8)
Tracheal infection	3 (4.2)
Outcomes	-
Deceased	28 (38.4)
Survived and discharged	43 (60.6)

**Table 5:** Comparison of tracheostomy-related events between survivors and non-survivors.

Tracheostomy Events	Mean ± SD Duration in Non-Survivors	Mean ± SD Duration in Survivors	*p*
Hospital admission to tracheostomy	12.75 ± 2.41	13.70 ± 2.28	0.533
Intubation to tracheostomy	10.18 ± 2.40	10.63 ± 2.7	0.176
ICU admission to tracheostomy	10.68 ± 2.07	11.05 ± 2.30	0.458
Tracheostomy to weaning from the ventilator	6.71 ± 1.68	6.19 ± 1.70	0.867
Tracheostomy to ICU discharge	12.32 ± 2.35	14.56 ± 3.69	0.044

## Data Availability

The information and data of this research are saved and stored digitally in a password-protected work-based computer, which can only be accessed by the investigators.

## References

[1] World Health Organization WHO Coronavirus (COVID-19) Dashboard. https://covid19.who.int.

[2] Lim C.-K., Ruan S.-Y., Lin F.-C., Wu C.L., Chang H.T., Jerng J.S., Wu H.D., Yu C.J. (2015). Effect of tracheostomy on weaning parameters in difficult-to-wean mechanically ventilated patients: A prospective observational study. PLoS ONE.

[3] Huang C., Wang Y., Li X., Ren L., Zhao J., Hu Y., Zhang L., Fan G., Xu J., Gu X. (2020). Clinical features of patients infected with 2019 novel coronavirus in Wuhan, China. Lancet.

[4] Ahmed Y., Cao A., Thal A., Shah S., Kinkhabwala C., Liao D., Li D., Parides M., Mehta V., Ow T. (2021). Tracheotomy Outcomes in 64 Ventilated COVID-19 Patients at a High-Volume Center in Bronx, NY. Laryngoscope.

[5] Johnson-Obaseki S., Veljkovic A., Javidnia H. (2016). Complication rates of open surgical versus percutaneous tracheostomy in critically ill patients. Laryngoscope.

[6] Mecham J.C., Thomas O.J., Pirgousis P., Janus J.R. (2020). Utility of Tracheostomy in Patients With COVID-19 and Other Special Considerations. Laryngoscope.

[7] Chiesa-Estomba C.M., Lechien J.R., Calvo-Henríquez C., Fakhry N., Karkos P.D., Peer S., Sistiaga-Suarez J.A., Gónzalez-García J.A., Cammaroto G., Mayo-Yánez M. (2020). Systematic review of international guidelines for tracheostomy in COVID-19 patients. Oral Oncol..

[8] Courtney A., Lignos L., Ward P.A., Vizcaychipi M.P. (2021). Surgical Tracheostomy Outcomes in COVID-19-Positive Patients. OTO Open.

[9] Chao T.N., Harbison S.P., Braslow B.M., Hutchinson C.T., Rajasekaran K., Go B.C., Paul E.A., Lambe L.D., Kearney J.J., Chalian A.A. (2020). Outcomes After Tracheostomy in COVID-19 Patients. Ann. Surg..

[10] Li X., Xu S., Yu M., Wang K., Tao Y., Zhou Y., Shi J., Zhou M., Wu B., Yang Z. (2020). Risk factors for severity and mortality in adult COVID-19 inpatients in Wuhan. J Allergy Clin. Immunol..

[11] Givi B., Schiff B.A., Chinn S.B., Clayburgh D., Iyer N.G., Jalisi S., Moore M.G., Nathan C.A., Orloff L.A., O’Neill J.P. (2020). Safety recommendations for evaluation and surgery of the head and neck during the COVID-19 pandemic. JAMA Otolaryngol.–Head Neck Surg..

[12] Takhar A., Walker A., Tricklebank S., Wyncoll D., Hart N., Jacob T., Arora A., Skilbeck C., Simo R., Surda P. (2020). Recommenda- tion of a practical guideline for safe tracheostomy during the COVID-19 pandemic. Eur. Arch. Otorhinolaryngol..

[13] David A.P., Russell M.D., El-Sayed I.H., Russell M.S. (2020). Tracheostomy guidelines developed at a large academic medical center during the COVID-19 pandemic. Head Neck.

[14] Mcgrath B.A., Brenner M.J., Warrillow S.J., Pandian V., Arora A., Cameron T.S., Añon J.M., Martínez G.H., Truog R.D., Block S.D. (2020). Tracheost- omy in the COVID-19 era: Global and multidisciplinary guidance. Lancet Respir. Med..

[15] Volo T., Stritoni P., Battel I., Zennaro B., Lazzari F., Bellin M., Michieletto L., Spinato G., Busatto C., Politi D. (2021). Elective tracheostomy during COVID-19 outbreak: To whom, when, how? Early experience from Venice, Italy. Eur. Arch. Oto-Rhino-Laryngol..

[16] Tang Y., Wu Y., Zhu F., Yang X., Huang C., Hou G., Xu W., Hu M., Zhang L., Cheng A. (2020). Tracheostomy in 80 COVID-19 Patients: A multicenter, retrospective, observational study. Front. Med..

[17] Avilés-Jurado F.X., Prieto-Alhambra D., González-Sánchez N., De Ossó J., Arancibia C., Rojas-Lechuga M.J., Ruiz-Sevilla L., Remacha J., Sánchez I., Lehrer-Coriat E. (2020). Timing, complications, and safety of tracheotomy in critically Ill patients with COVID-19. JAMA Otolaryngol.–Head Neck Surg..

[18] Adly A., Youssef T.A., El-Begermy M.M., Younis H.M. (2018). Timing of tracheostomy in patients with prolonged endotracheal intubation: A systematic review. Eur. Arch. Otorhinolaryngol..

[19] Wang R., Pan C., Wang X., Xu F., Jiang S., Li M. (2019). The impact of tracheotomy timing in critically ill patients undergoing mechanical ventilation: A meta-analysis of randomized controlled clinical trials with trial sequential analysis. Heart Lung.

[20] Chandran A., Kumar R., Kanodia A., Shaphaba K., Sagar P., Thakar A. (2021). Outcomes of Ttracheostomy in COVID-19 Patients: A Single Centre Experience. Indian J. Otolaryngol. Head Neck Surg..

[21] Thal A.G., Schiff B.A., Ahmed Y., Cao A., Mo A., Mehta V., Smith R.V., Cohen H.W., Ow T.J. (2021). Tracheotomy in a high-volume center during the COVID-19 pandemic: Evaluating the surgeon’s risk. Otolaryngol. Head Neck Surg..

[22] Zhou F., Yu T., Du RFan G., Liu Y., Liu Z., Xiang J., Wang Y., Song B., Gu X. (2020). Clinical course and risk factors for mortality of adult inpatients with COVID-19 in Wuhan, China: A retrospective cohort study. Lancet.

